# Synthesis of Sandwiched Composite Nanomagnets by Epitaxial Growth of Fe_3_O_4_ Layers on SrFe_10_Cr_2_O_19_ Nanoplates in High-Boiling Organic Solvent

**DOI:** 10.3390/nano13010167

**Published:** 2022-12-30

**Authors:** Evgeny O. Anokhin, Danila A. Deyankov, Zitian Xia, Ekaterina S. Kozlyakova, Vasily A. Lebedev, Anatolii V. Morozov, Daniil A. Kozlov, Roy R. Nygaard, Dmitry I. Petukhov, Lev A. Trusov

**Affiliations:** 1 Faculty of Chemistry, Lomonosov Moscow State University, Moscow 119991, Russia; 2 Faculty of Materials Science, Lomonosov Moscow State University, Moscow 119991, Russia; 3 Faculty of Materials Science, MSU-BIT University, Shenzhen 518172, China; 4 Faculty of Physics, Lomonosov Moscow State University, Moscow 119991, Russia; 5 Functional Quantum Materials Laboratory, National University of Science and Technology «MISiS», Moscow 119049, Russia; 6 Bernal Institute, University of Limerick, Limerick V94 T9PX, Ireland; 7 Skolkovo Institute of Science and Technology, Moscow 121205, Russia; 8 Kurnakov Institute of General and Inorganic Chemistry of the Russian Academy of Sciences, Moscow 119991, Russia

**Keywords:** permanent magnets, hexaferrites, magnetic nanocomposites, nanomagnets, epitaxy, exchange coupling, shape anisotropy

## Abstract

Herein, we demonstrate the synthesis of sandwiched composite nanomagnets, which consist of hard magnetic Cr-substituted hexaferrite cores and magnetite outer layers. The hexaferrite plate-like nanoparticles, with average dimensions of 36.3 nm × 5.2 nm, were prepared via a glass crystallization method and were covered by spinel-type iron oxide via thermal decomposition of iron acetylacetonate in a hexadecane solution. The hexaferrite nanoplates act as seeds for the epitaxial growth of the magnetite, which results in uniform continuous outer layers on both sides. The thickness of the layers can be adjusted by controlling the concentration of metal ions. In this way, layers with an average thickness of 3.7 and 4.9 nm were obtained. Due to an atomically smooth interface, the magnetic composites demonstrate the exchange coupling effect, acting as single phases during remagnetization. The developed approach can be applied to any spinel-type material with matching lattice parameters and opens the way to expand the performance of hexaferrite nanomagnets due to a combination of various functional properties.

## 1. Introduction

Nanoparticle magnets should combine several important inherent properties. Firstly, they must maintain a constant magnetic moment, even at very small particle sizes; the material must demonstrate non-zero residual magnetization and coercive force. This allows the nanomagnets to create a magnetic field around themselves and interact with external magnetic fields in such a way that it is possible to control not only their position but also their orientation. Secondly, the materials must be chemically and thermally stable so as not to degrade in the environment. Thirdly, it should be possible to integrate them with other materials to create composite nanomagnets combining various functional characteristics. Therefore, not many substances are known that can be used to design nanomagnets. Metal-based magnetics (i.e., FePt, FeCo, Nd-Fe-B and other rare-earth alloys) demonstrate superior magnetic characteristics; however, they are highly reactive at the nanoscale [1,2]. The spinel ferrites (Fe_3_O_4_, γ-Fe_2_O_3_, CoFe_2_O_4_, etc.), which are commonly used to produce ferrofluids, lose their remanence and coercivity when particle sizes decrease due to superparamagnetism [3]. Among magnetically hard ferrites, two possible candidates are known, which are ε-Fe_2_O_3_ and M-type hexaferrites (MFe_12_O_19_, M = Ba, Sr). Epsilon iron oxide reveals very high magnetocrystalline anisotropy and remains hard-magnetic down to the nanoscale [2,4,5]. However, this polymorphic modification is metastable, and to date, there are no methods for efficient production of this phase with controllable particle morphologies. Currently, the best-known methods for producing ε-Fe_2_O_3_ mainly include high-temperature crystallization in a silica matrix followed by its removal and usually leads to the production of non-single-phase powders contaminated by impurity phases, such as γ-, α- and β-iron oxide polymorphs, and also some amount of residual SiO_2_ on the particle surface [2,6,7]. To our knowledge, there are also no reports on the successful introduction of ε-Fe_2_O_3_ into complex nanoparticle magnets.

Hard magnetic hexaferrites are currently available materials for creating composite nanomagnets. Hexaferrite particles retain high coercivity down to sizes of about 10 nm, the material is thermally and chemically stable, and there are many various methods for producing hexaferrites, including well-developed industrial routes [8,9]. Moreover, the hexaferrite nanoparticles are usually plate-like, and such anisotropy generates additional effects of self-organization, magnetic, and magnetooptical properties [10,11,12,13,14,15]. The main challenge to producing hexaferrite-based nanocomposites is the need to obtain individual non-aggregated hexaferrite nanoparticles. Most methods of hexaferrite synthesis involve high-temperature processing, which leads to aggregation and sintering of the particles. On the other hand, low-temperature processing often leads to phase impurity, low crystallinity of the material, a large number of defects and, consequently, reduced magnetic characteristics. In addition, even non-sintered nanoparticles tend to aggregate due to strong magnetic attraction, so colloidal stabilization is needed to cover each particle individually by other phases.

The glass-ceramic technique is a suitable method for creating hard magnetic hexaferrite cores for composite nanomagnets [16,17]. Unlike other production methods, it obtains non-aggregated hexaferrite nanoparticles of high structural quality. The method makes it possible to additionally adjust the morphology of the formed particles by varying the composition and glass crystallization conditions [18], as well as to carry out elemental substitutions in the hexaferrite structure, which result in an adjustment of the magnetic characteristics [19]. Previously, we reported the preparation of chromium-substituted hexaferrite nanoplates by glass crystallization, which resulted in record values of the coercivity among nanosized hexaferrite particles [20]. Such particles are very promising for application as hard magnetic cores for composite nanomagnets.

Modifying the surface of each hexaferrite particle individually with different materials is not an easy task due to the interparticle attraction and irreversible adhesion. Only a few examples are known when hexaferrite particles were coated from stable colloidal solutions, e.g., amorphous SiO_2_ shells prepared by fast hydrolysis of tetraethoxysilane [21] and exchange-coupled sandwiched composite nanoparticles with spinel ferrite layers (γ-Fe_2_O_3_, ZnFe_2_O_4_ and MnFe_2_O_4_) produced by co-precipitation from aqueous solutions [9,22,23,24]. Recently, we have developed a method for the direct deposition of CoFe_2_O_4_ outer layers on hexaferrite particle surfaces via the decomposition of metal-organic salts in high-boiling solvents. It was shown that spinel ferrite grows epitaxially on the hexaferrite surface, continuing the spinel block of the hexaferrite crystal structure. It has been suggested that layers of other spinel phases can be grown in this way, and the thickness of the layers can be adjusted by changing the concentration of the initial salts in the solution.

Herein we report the synthesis of sandwiched nanoparticles based on chromium-substituted strontium hexaferrite as cores of the composite and outer layers of epitaxially grown magnetite Fe_3_O_4_. High-quality hexaferrite nanoplates were obtained using the glass crystallization method, and the spinel layers of various thicknesses were produced by iron acetylacetonate thermal decomposition in a hexadecane solution.

## 2. Materials and Methods

### 2.1. Materials

Strontium carbonate SrCO_3_ (≥99.9%), iron (III) oxide Fe_2_O_3_ (<5 μm, ≥99%), chromium (III) oxide Cr_2_O_3_ (≥98%), boric acid H_3_BO_3_ (≥99.8%), oleylamine (cis-1-amino-9-octadecene, technical grade, 70%), oleic acid C_17_H_33_COOH (90%), iron (III) acetylacetonate Fe(C_5_H_7_O_2_)_3_ (≥97%), hexane C_6_H_14_ (≥97.94%), hexadecane C_16_H_34_ (≥98.0%). All from Sigma-Aldrich (St. Louis, MO, USA).

### 2.2. Hexaferrite Nanoparticles Synthesis

Individual strontium hexaferrite nanoparticles were obtained by the oxide glass crystallization technique described in ref. [20]. Briefly, for the preparation of glass with a nominal composition of 25SrO-4Fe_2_O_3_-2Cr_2_O_3_-12B_2_O_3_, precursors (SrCO_3_, Fe_2_O_3_, Cr_2_O_3_, and H_3_BO_3_) were mixed in a stoichiometric ratio and then melted in a platinum crucible in a high-temperature furnace. The mixture was heated up to 1350 °C at a rate of about 35 °C/min and then exposed to that temperature for 1 h. The resulting melt was quenched between rotating steel rollers into the water to form glassy flakes. The obtained glass was isothermally annealed at 750 °C for 2 h to crystallize hexaferrite nanoparticles. The resulting glass-ceramic sample, consisting of strontium hexaferrite and a borate matrix, was ground in an agate mortar. The obtained powder was treated with 3% hydrochloric acid to dissolve the borate matrix and extract the hexaferrite particles. Hydrochloric acid was added to the powder and the mixture was sonicated for 10 min with simultaneous heating to 40 °C. After that, the magnetic particles were separated by a magnet, then the remaining powder was again washed in acid until the non-magnetic matrix was completely removed. The precipitate obtained after the magnetic separation and decantation was dispersed in distilled water. Then the particles were separated by centrifugation, and the powder was dried in a drying box for 30 min at 120 °C. The obtained raw hexaferrite sample is labeled as SHF in this manuscript.

### 2.3. Hexaferrite/Magnetite Composites Synthesis

Two composite samples with different hexaferrite-to-iron oxide ratios were prepared. The samples were labelled FO_32 and FO_11, corresponding to nominal volume proportions SrFe_12_O_19_:Fe_3_O_4_ equal to 3:2 and 1:1, which were estimated from the maximum possible iron oxide content.

The synthesis of the composites was carried out by thermal decomposition of iron (III) acetylacetonate Fe(C_5_H_7_O_2_)_3_ under an inert atmosphere in hexadecane acting as a high-boiling solvent (boiling point at 287 °C [25]). The strontium hexaferrite powder (100 mg for each sample) and iron acetylacetonate (591 and 887 mg for samples FO_32 and FO_11, respectively) were added to 40 mL of hexadecane. Furthermore, 2 mL of oleic acid and oleylamine (again, 2.6 and 4 mL for samples FO_32 and FO_11) were added both acting as surfactants and reducing agents. The mixture of these components was sonicated for 30 min at room temperature to disperse the particles, then it was placed in the three-neck flask (necks for thermocouple, Ar inlet, and mechanical stirrer). Argon flow of 150 mL/min for 30 min was used to remove air from the flask and then was kept during the synthesis. After that, the solution was heated to 270 °C and exposed at this temperature for 30 min with continuous stirring. The flask with the solution was then quickly cooled to room temperature, after which the Ar flow was stopped.

The composite particles were magnetically separated from the reaction mixture, and then washed several times alternately with increasing polarity: hexane, acetone, ethanol, 1 M sodium hydroxide solution, and distilled water until the behavior of the powder changed to hydrophilic to remove surfactant residues.

### 2.4. Characterization of Samples

Powder X-ray diffraction studies (XRD) were performed using a Rigaku (Tokyo, Japan) D/MAX 2500 diffractometer (Cu Kα radiation) at room temperature. The full-profile analysis of the patterns was carried out by the Rietveld method using MAUD software (ver. 2.99) [26] (Trento, Italy). The instrumental broadening was calculated using the Y_2_O_3_ standard.

The inductively coupled plasma mass spectrometry method (ICP-MS) was used to determine the chemical composition of the magnetic composites. The analysis was carried out using a PerkinElmer (Waltham, MA, USA) Elan DRC II instrument. The powders were dissolved in an aqua regia for ICP-MS sample preparation.

For the transmission electron microscopy (TEM) investigation, a tiny amount of the powder sample was dispersed in ethyl alcohol, and then one drop of the suspension was deposited onto a carbon film supported by a copper grid. Transmission electron microscopy was performed using a 200 kV field emission microscope JEOL (Tokyo, Japan) 2100 F in a bright-field mode. For magnifications 100 kX and lower, the objective aperture was in use to contrast images. To determine the average particle diameter, more than 400 particles were counted for each sample, and for the particle thickness, we used at least 66 particles for each sample. Mean particle dimensions and standard deviations were obtained by fitting TEM histograms with a lognormal distribution function. Selected area electron diffraction (SAED) patterns integration and background elimination were carried out using CrysTBox software (ver. 1.10) [27,28] (Prague, Czech Republic). Atomic resolution high-angle annular dark field scanning transmission electron microscopy (HAADF-STEM) images as well as energy-dispersive X-ray (EDX) maps were acquired on a probe aberration-corrected ThermoFisher (Waltham, MA, USA) Titan Themis Z electron microscope at 200 kV equipped with a Super-X system for EDX analysis.

Magnetic measurements in the maximum field strength of 30 kOe and at temperatures of 5, 100, 200, and 300 K were carried out using a Vibrating Sample Magnetometer (VSM) as part of the PPMS-9T Physical Property Measurement System (Quantum Design, San Diego, CA, USA). Powder samples were fixed with a polymer varnish to avoid their movement in the magnetic field.

The Curie temperature of the magnetic composites was determined using a PerkinElmer (Waltham, MA, USA) Pyris Diamond TG/DTA thermal analyzer in the field generated by a permanent neodymium magnet. The magnet was suspended above the sample being heated and cooled, so a mass gain was observed during the transition to the demagnetized state and vice versa. The rate of heating and cooling during measurements was 10 °C/min; the samples were cyclically heated and cooled in the temperature range of 100–700 °C. Prior to measurement, the sample was magnetized in the applied magnetic field of the magnet by heating to a temperature of 400 °C and cooling in the field. The Curie points were defined as the intersection points of the tangents to the curves before and after the transition.

## 3. Results and Discussion

**Figure 1:**
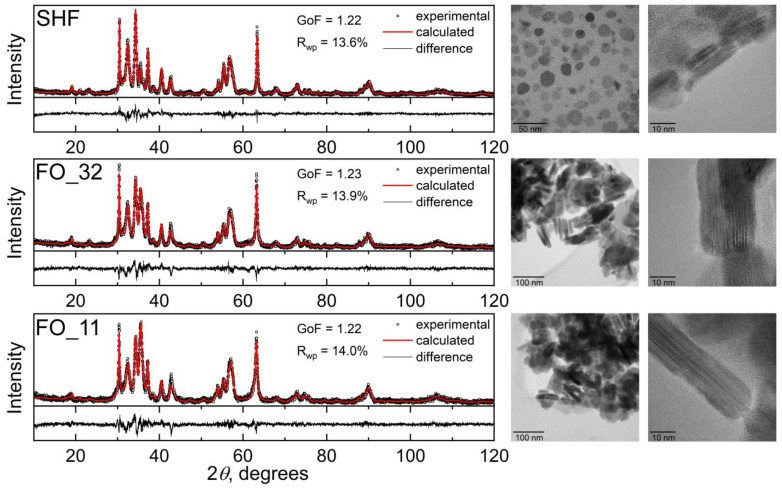
X-ray diffraction patterns (**left**) and transmission electron microscopy images (**right**) of the hexaferrite and composite samples.

**Figure A1:**
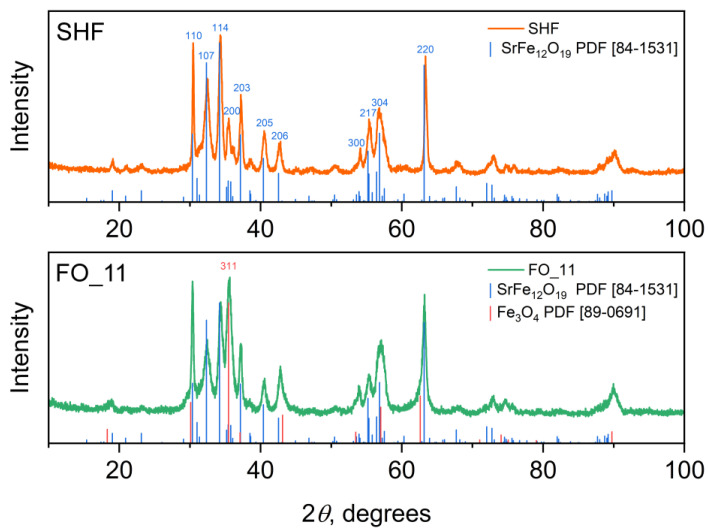
XRD patterns of SHF and FO_11 samples. Royal blue and coral columns represent hexaferrite and magnetite reflections. Note that only (311) Fe_3_O_4_ line does not coinside hexaferrite peaks.

According to XRD (Figure 1, left), the raw hexaferrite sample SHF is a single-phase M-type hexaferrite (space group *P*6_3_/*mmc*) with the unit cell parameters *a* = 5.8710(5) Å and *c* = 23.012(3) Å. The parameters are slightly reduced in comparison with undoped SrFe_12_O_19_ (*a* = 5.885 and *c* = 23.05(3) Å [29]), due to a substitution of some iron ions by chromium [20], which has a smaller ionic radius (*r*^IV^(Fe^3+^) = 0.645 Å and *r*^IV^(Cr^3+^) = 0.615 Å [30]). A noticeable broadening of the diffraction lines indicates the small size of the particles; an even stronger broadening of the *hk0* reflections (Figure A1 from Appendix A) indicates the shape anisotropy of the particles with a smaller particle dimension along the crystallographic direction *c*, that is, the plate-like shape.

**Figure A2:**
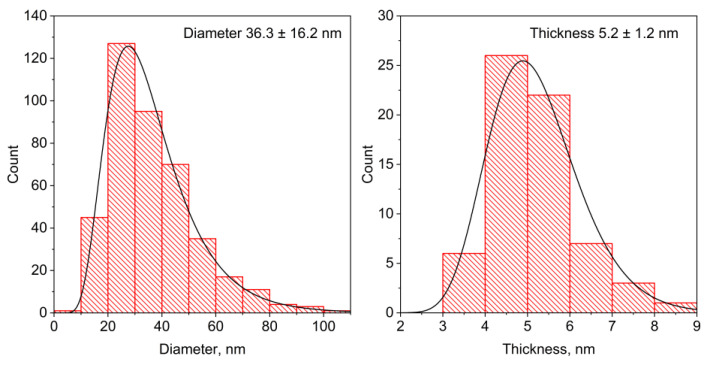
Particle diameter and thickness distributions of SHF sample obtained from TEM data.

**Figure A3:**
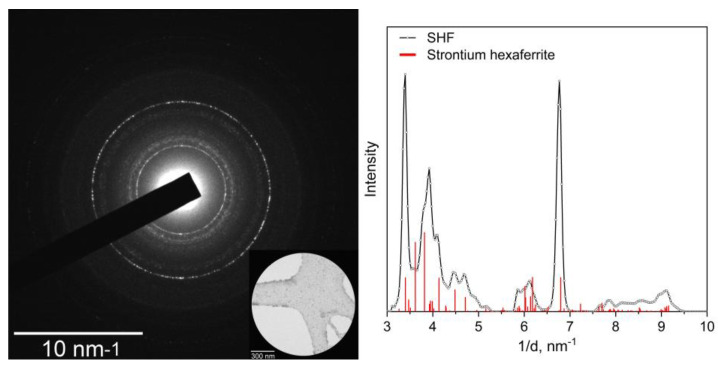
Selected area electron diffraction pattern (inset shows the selected area) and radial-integrated profile of SHF sample.

The morphology of the raw hexaferrite particles was also investigated using transmission electron microscopy (TEM) (Figure 1, right). According to TEM, SHF particles are thin anisotropic plates with an average diameter of 36.3 nm and an average thickness of 5.2 nm (Figure A2 from Appendix A), which is consistent with the observation of the anisotropic XRD line broadening. In addition, the particle dimensions are estimated by the XRD line broadening analysis, resulting in 30 nm and 6 nm for the estimated average thickness and diameter, correspondingly. A comparison of the integration of the selected area electron diffraction (SAED) pattern with hexaferrite diffraction maxima confirmed that the nanoparticles on the image possessed the strontium hexaferrite structure (Figure A3 from Appendix A). The chemical analysis of the raw sample resulted in a Sr:Fe:Cr ratio equal to 0.9:10:2. The reduced strontium content in hexaferrite nanoparticles compared with the bulk state was previously reported, and it is due to the limited number of atomic layers in the *c*-direction and preferential spinel blocks on the particle surfaces, which do not contain strontium [20,31,32].

According to XRD, the samples FO_32 and FO_11 are two-phase composites consisting of strontium hexaferrite and spinel iron oxide, i.e., magnetite Fe_3_O_4_ or maghemite γ-Fe_2_O_3_. These spinel phases possess very similar soft magnetic properties, as well as identical crystalline structures, and even form a continuous solid solution; thus, it is difficult to accurately distinguish them, especially at the nanoscale. The calculated lattice parameter of the spinel phase is a = 8.370(1) Å for each sample, which is smaller than that of magnetite (a = 8.387 Å, PDF 89-0691) and larger than that of maghemite (a = 8.352 Å, PDF 39-1346). Taking into account the reducing atmosphere during the synthesis, the spinel phase was considered as magnetite Fe_3_O_4_. M-type hexaferrite and magnetite match structurally in a certain crystallographic direction, due to the presence of the spinel-structured block in the hexaferrite unit cell. In the X-ray diffraction patterns of the composite samples (Figure 1 and Figure A1 from Appendix A), the peak at 35.5 degrees, which corresponds to the most intense peak of Fe_3_O_4_, significantly rises, indicating the presence of magnetite. The patterns are well fitted by these two phases, and the phase content can also be estimated (Table 1). To more accurately determine the excess of the iron oxide phase compared with the initial hexaferrite powder, the chemical composition of the samples was investigated using ICP-MS (Table 1). The mass fraction of Fe_3_O_4_ (ICP-MS) was calculated considering the additional amount of iron relative to chromium, assuming that the ratio of chromium to iron in the hexaferrite cores did not change during the synthesis. According to the analysis, the mass fraction of the spinel iron oxide increases with an increase in the amount of iron acetylacetonate used during the synthesis; however, it is lower than the nominal values. The decreased amount of Fe_3_O_4_ relative to the nominal composition can be explained by an incomplete chemical reaction during the synthesis.

**Figure 2:**
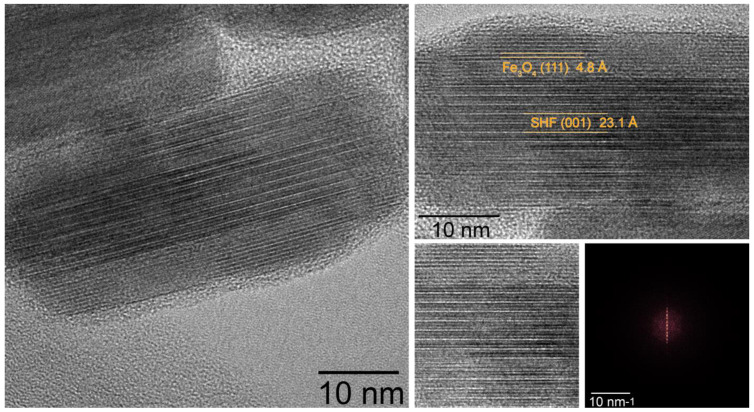
HRTEM image of FO_11 sample composite particle. Right images are cropped parts of the left one; at the right bottom side of the collage, 2D FFT of the cropped square region is presented.

**Figure 3:**
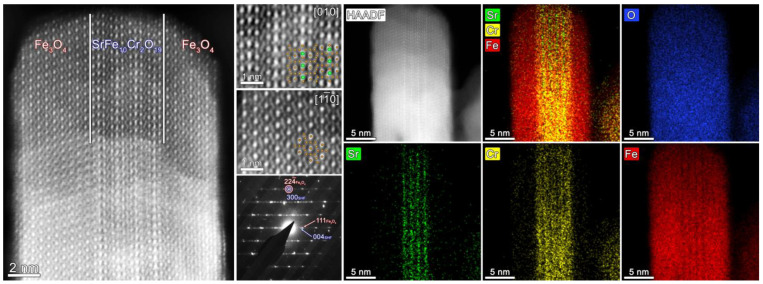
High-resolution HAADF STEM images: overall view of the Fe_3_O_4_/SrFe_10_Cr_2_O_19_/Fe_3_O_4_ composite sample FO_11; inner hexaferrite and outer spinel parts (green balls represent Sr, brown—Fe/Cr), SAED pattern of the area. At the right part of the collage HAADF-STEM image of the composite particle and color-coded EDX elemental (Sr, Cr, Fe, O) maps are presented.

Transmission electron microscopy (TEM) (Figure 1, right) showed that the composite samples consist of well-defined sandwich-like particles. Hexaferrite plates are in the center of the composite particles, and magnetite layers are formed on top of them by heterogeneous nucleation, while free magnetite nanoparticles are not formed at all in the volume of the solution. The sandwiched structure is visible when the particles are oriented edge-on (Figure 2 and Figure 3). The magnetite grows symmetrically and uniformly producing well-crystalline layers of the same thickness on both sides. The lateral sides of the hexaferrite cores are free of magnetite, which indicates that the seeded growth of the spinel iron oxide occurs only on the spinel blocks of the hexaferrite structure; thus, the magnetite layers continue the hexaferrite structure.

According to the results of HR-TEM with 2D FFT (Figure 2), HAADF STEM and SAED (Figure 3) the epitaxial relations between Fe_3_O_4_ and SrFe_10_Cr_2_O_19_ phases are the following:

$$\{\begin{array}{c} \left(111\right){\mathrm{Fe}}_{3}O_{4}//\left(001\right)Sr{\mathrm{Fe}}_{10}{\mathrm{Cr}}_{2}O_{19}\\ \left[11\bar{2}\right]{\mathrm{Fe}}_{3}O_{4}//\left[100\right]Sr{\mathrm{Fe}}_{10}{\mathrm{Cr}}_{2}O_{19}. \end{array}$$

**Figure 4:**
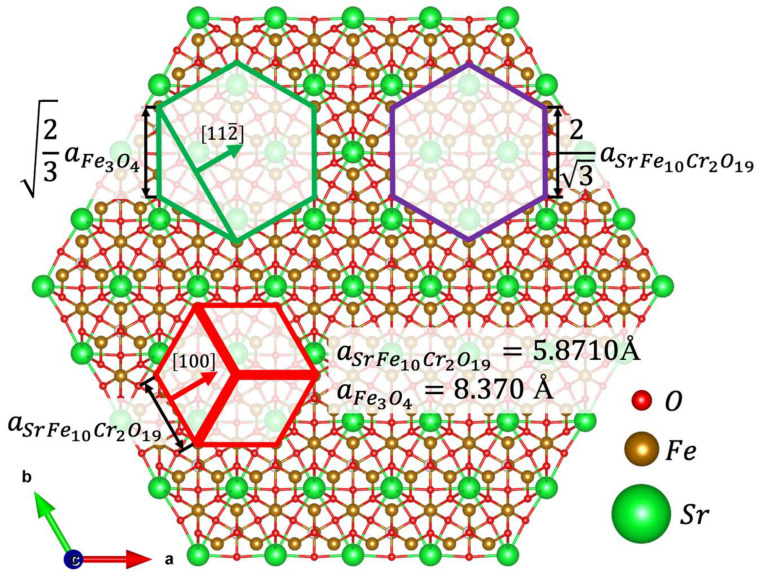
The atomic structure of the terminal SrFe_10_Cr_2_O_19_ layer which participates in the formation of the interface with Fe_3_O_4_. The most favorable position of Fe_3_O_4_ unit cell, SrFe_10_Cr_2_O_19_ unit cell and a coincide cite lattice cell are marked as green, red, and purple hexagons respectively. Lengths of hexagon sides are presented in the lattice parameter units [34].

The results of the MD simulations are based on the algorithm reported in ref. [33] allowed us to propose the model of the interface that had formed between the Fe_3_O_4_ and SrFe_10_Cr_2_O_19_ phases (Figure 4). It should be noted that both SAED and simulation results yield the epitaxial relations that were stated above. At first glance, it might seem unusual that Fe_3_O_4_ and SrFe_10_Cr_2_O_19_ unit cells are 30 degrees rotated with respect to each other (green and red hexagons, respectively). However, a closer look at the HAADF HRTEM image (Figure 3) reveals that spinel iron oxide has formed on a surface which is structurally ideal for the growth of this phase, and the rotation is only apparent; the spinel-structured iron oxide continues the spinel structural block of the SrFe_10_Cr_2_O_19_ unit cell in the [111] direction (of the cubic cell). Based on the proposed model, we have estimated the lattice mismatch between the Fe_3_O_4_ (111) layer and the ideal coincide site lattice—CSL (green and purple hexagons respectively). It should be noted that in the classical formula that is commonly used to calculate the lattice mismatch between substrate and film, it is principal which phase is being considered as the earlier and which one as the latter since the sign in front of the mismatch value provides information as to whether the film is being stretched or compressed by the substrate. In our case, we decided to consider SrFe_10_Cr_2_O_19_ as a substrate since these nanoparticles were introduced into the high-boiling solvent to promote the epitaxial growth of Fe_3_O_4_. The mismatch value, calculated using the formula:

$$\varepsilon =\frac{\sqrt{\frac{2}{3}}a_{Fe_{3}O_{4}}-\frac{2}{\sqrt{3}}2a_{SrFe_{10}Cr_{2}O_{19}}}{\frac{2}{\sqrt{3}}2a_{SrFe_{10}Cr_{2}O_{19}}}\cdot 100\%=0.81\%$$

is quite low, which is in reasonable agreement with the experimental results. It occurs that in the case of the synthesized nanoparticles the spinel iron oxide is slightly compressed in the interface plane.

EDX elemental maps (Figure 3) show that the hexaferrite core remains unchanged after synthesis: strontium remains in its sublattice, and chromium is absent in the outer magnetite layers.

**Figure A4:**
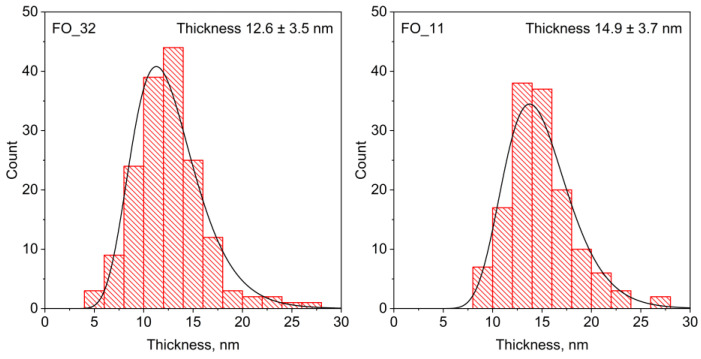
Particle thickness distributions of FO_32 and FO_11 composite samples obtained from TEM data.

The thickness of the composite sandwiches noticeably differs from the thickness of the initial particles, and it increases with the rise in the iron oxide content: 12.6 and 14.9 nm for the samples FO_32 and FO_11, respectively (Figure A4 from Appendix A). By subtracting the average thickness of the original particles, the thickness of the coating of the sandwich composites can be estimated. The average thickness of magnetite, grown on each side of the hexaferrite plate-like cores, is about 3.7 and 4.9 nm for samples FO_32 and FO_11, respectively. The advantage of the developed method for the controllable formation of very thin as well as rather thick spinel layers upon hexaferrite nanoparticles is high crystal quality and uniformity compared with low-temperature water solution-based methods [23,24,35]. Furthermore, the reaction at high temperatures promotes the coating of each particle, since when heated, the magnetization of hexaferrites decreases sharply and particles cease to magnetize to each other. This makes it possible to successfully cover particles that have high magnetization at room temperature.

The initial hexaferrite nanoparticles are strongly hard-magnetic. Their room-temperature coercivity of 6350 Oe is one of the highest reported for hexaferrite particles of such a small size [8,10,18,20,32,36]. This is mainly due to the partial chromium substitution [20]. The magnetization is also larger than is typically observed in hexaferrite nanoparticles, in which the saturation magnetization is reduced because of low crystallinity, rising surface effects, and non-collinear spin orientation [8,32]. The crystal quality of the hexaferrite particles is provided via a glass crystallization synthesis method. During cooling, the coercive force of the material practically does not change, and the magnetization increases as expected.

**Figure 5:**
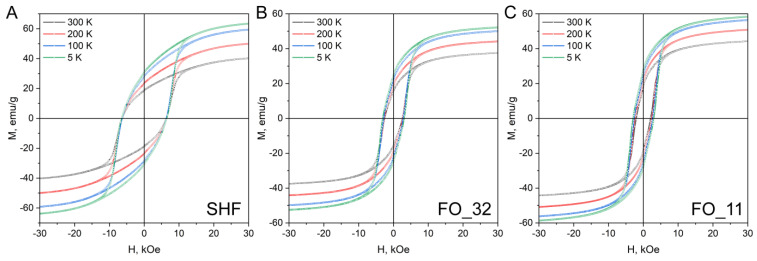
Hysteresis loops of the uncovered SrFe_10_Cr_2_O_19_ (SHF) nanoparticles and composite samples (FO_32 and FO_11) at different temperatures (5, 100, 200 and 300 K).

**Figure 6:**
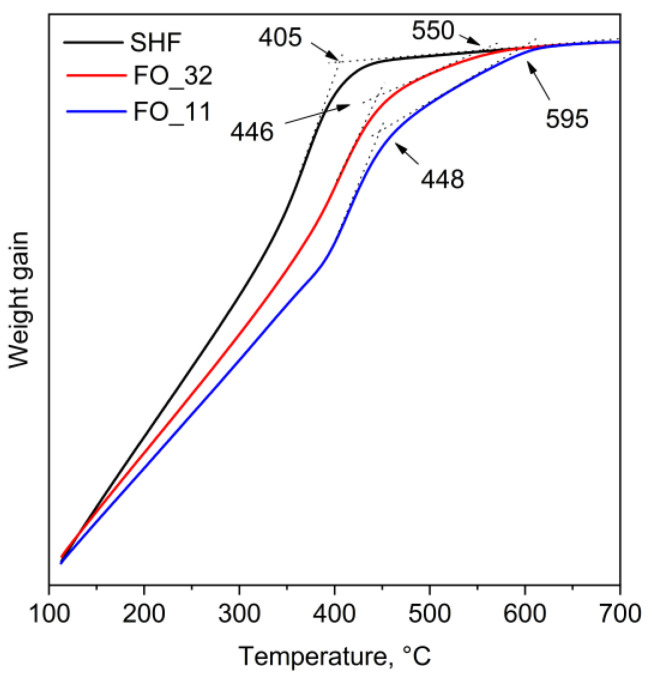
Thermogravimetry curves for raw SrFe_10_Cr_2_O_19_ (SHF) and composite samples (FO) measured in an applied magnetic field.

The hysteresis loops of the composite samples (Figure 5) do not possess any signs of separate soft and hard magnetic material mixtures, i.e., there are no additional inflection points or curvature deviations from single-phase hysteresis in the entire investigated range of temperatures. This may indicate that all samples behave as single magnetic phases and not as a mixture of magnetically hard and magnetically soft phases. It is generally considered that the soft magnetic phase rigidly coupled to the magnetically hard phase is twice the width of a domain wall in the hard phase [37]. For M-type hexaferrites, the width of the domain wall is approximately 14 nm [9]; thus, the magnetite phase should be coupled to the hexaferrite core. The additional inflection point would have been observed on the hysteresis loops in the absence of magnetic exchange coupling between the phases [24]. Thus, the hysteresis shape proves that the composite samples do not contain any significant amount of non-coupled magnetite. Even though the two phases in the composites act as a single magnetic phase during remagnetization, the Curie point measurements (Figure 6) show two temperatures of the magnetic transitions: about 450 °C for the hexaferrite cores (405 °C for uncovered SrFe_10_Cr_2_O_19_ nanoparticles) as well as 550 and 595 °C for the magnetite layers in the samples FO_32 and FO_11, respectively [8,24,38]. It is also clearly seen that sample FO_11 contains a larger amount of Fe_3_O_4_ than sample FO_32, which is indicated by a higher mass loss during cooling below *T_C_*. It is consistent with the results of the chemical analysis. The Curie temperature rise for both magnetite and hexaferrite phases in the composite particles (Table 2) could be caused by structural distortions of crystal lattices and cohesive energy changes. The structural distortions, i.e., the variation of bond angles and lengths, led to changes in magnetic exchange interactions in both phases. The cohesive energy is determined by bonding energies, so it greatly depends on the surface effects in the nanoparticles and their shape as well as on the crystal structure tensions and distortions caused by the influence of the epitaxially grown layers [39]. The observed effects of Curie temperature shift can be explained by these changes in magnetic exchange interactions and cohesive energy; however, further detailed research is needed.

The magnetic characteristics of the samples are shown in Table 3. The deposition of the soft-magnetic spinel iron oxide onto the hard-magnetic hexaferrite core has a small effect on saturation magnetization. In the sample FO_32, the magnetization even slightly decreases, and in the sample FO_11, it slightly rises. This may be due to size effects when thinner layers have reduced magnetization compared with bulk material. The coercivity of the composites is reduced to 2520 and 2020 Oe for the samples FO_32 and FO_11, respectively. Nevertheless, it should be noted that the obtained values of both magnetization and coercivity of the composite samples exceed those reported before for the exchange-coupled hexaferrite composites [22,24].

The coercivity *H*_C_ of uncovered SrFe_10_Cr_2_O_19_ nanoparticles remains almost unchanged and increases monotonically for both composite samples during cooling, which is quite unusual for hexaferrite materials [38]. For submicron single-domain particles, this behavior is described by the Stoner-Wohlfarth model, in which

$$H_{C}\left(T\right)\;\sim \;\frac{K_{1}\left(T\right)}{M_{S}\left(T\right)}$$

where *K*_1_ is the constant of the magnetocrystalline anisotropy, and *M_S_* is the saturation magnetization. Since *K*_1_ decreases slower than *M*_S_ with increasing temperature, the coercivity for large single-domain hexaferrite particles has a maximum above the room temperature (~400 K), i.e., in the range of 5–300 K the coercivity increases. For nanoparticles, the coercivity is determined not only by the magnetocrystalline anisotropy but also by the significant contribution of the surface anisotropy

$$K_{eff}=K_{vol}+6\frac{K_{S}}{d}$$

where *K_eff_* is the effective constant of the magnetocrystalline anisotropy, *K_vol_* is the magnetocrystalline anisotropy constant for a bulk sample, *K_S_* is the constant of the surface anisotropy, and *d* is the diameter of a spherical particle. In this case, for hexaferrite, the surface contribution significantly reduces the effective anisotropy. As the particle size decreases, the surface contribution increases, which leads to a decrease in the coercivity [16]. The surface contribution is assumed as a non-collinear distribution of spins on the particle surface [40]. It is believed that with a decrease in temperature, the thickness of this distorted subsurface layer reduces, which leads to a decrease in the contribution of the surface to the magnetic anisotropy and, accordingly, to an increase in the coercive force.

The shape of the hysteresis loop for the SHF sample with the *M*_R_/*M*_S_ ratio close to 0.5 is similar to that described by the Stoner-Wohlfarth model of randomly oriented single-domain particles with uniaxial anisotropy [38]. For both composite samples, the *M*_R_/*M*_S_ ratio is close to 0.41 at room temperature, while for previously reported materials, it is significantly lower. This indicates that an easy magnetization axis direction is mainly determined by the hexaferrite core. Considering both magnetocrystalline and shape anisotropy, the exchange-coupled magnetic system can result in a somewhat tilted easy axis [41], which affects the *M*_R_/*M*_S_ ratio and leads to a change in the shape of the hysteresis loop. This ratio gradually increases up to 0.48 for the composite samples upon cooling to 5 K, indicating the changes occurring in the magnetic system of the nanocomposites.

## 4. Conclusions

In summary, for the first time, we have utilized high-quality Cr-substituted hexaferrite nanoplates as cores of sandwiched composite nanomagnets. The thin outer layers of magnetite Fe_3_O_4_ were epitaxially grown via the decomposition of organic salts in the hexadecane solution containing well-dispersed hexaferrite nanoparticles. The synthesis in the high-boiling solvent revealed two key advantages over the water-based co-precipitation methods. Firstly, at elevated temperatures, the magnetic moments of the hexaferrite particles sharply reduce, which promotes the formation of colloids of non-aggregated particles. This covers each particle individually. Secondly, the growth of magnetite at higher temperatures resulted in uniform continuous layers with high crystallinity. We have shown that the thickness of the layers can be adjusted by changing the concentration of metal ions. While the core hexaferrite particles had an average diameter of 36.3 nm and thickness of 5.2 nm, the magnetite layer thickness varied from 3.7 nm to 4.9 nm.

The outer Fe_3_O_4_ layers continue the spinel block of the hexaferrite structure; thus, the [111] axis of magnetite is co-directional with the [001] axis of the hexaferrite lattice. Therefore, the hexaferrite cores act as a template for the formation of quite unusual plate-like morphology of the spinel phase. The developed approach can be expanded to any spinel-type material with matching lattice parameters, while the parameters of the hexaferrite can be tuned by ionic substitution. This paves the way for the design of multifunctional nanomagnets, in which the hard magnetic properties of the hexaferrite cores and their possibility to align in a magnetic field are combined with optical, catalytic, ferroelectric, etc., properties of the outer layers. In our case, the epitaxial interface between hard and soft magnetic phases resulted in an exchange coupling effect, causing this two-phase composite to act as a single magnetic phase. This makes it possible to create new nanomagnets with synergistically combined properties which cannot be observed in the source materials.

## Figures and Tables

**Table 1:** Magnetite content ω determined by chemical analysis of the samples (ICP-MS) and by the Rietveld refinement of diffraction patterns. Chemical composition is normalized to (Fe + Cr) = 12 for the initial hexaferrite sample.

Sample	Fe:Cr Ratio (ICP-MS)	ω (Fe_3_O_4_), wt % (ICP-MS)	ω (Fe_3_O_4_), wt % (XRD)	ω (Fe_3_O_4_), wt % (Nominal)
SHF	10.0:2.0	0	0	0
FO_32	14.6:2.0	27.1	27.5	40
FO_11	18.2:2.0	39.8	37.5	50

**Table 2:** Curie temperatures of the samples measured by thermogravimetry in a magnetic field.

Sample	Curie Temperature *T_C_*, °C (K)	
SHF	405 (678)	—
FO_32	446 (719)	550 (823)
FO_11	448 (721)	595 (868)

**Table 3:** Magnetic properties of the uncovered SrFe_10_Cr_2_O_19_ (SHF) nanoparticles and composite samples (FO_32 and FO_11) at different temperatures. *H*_C_ represents coercivity, *M*_S_—sample magnetization at 30 kOe, *M*_R_—remanent magnetization.

Sample	Temperature, K	300	200	100	5
SHF	*H*_C_, Oe	6350	6430	6320	6450
	*M*_S_, emu/g	40.2	50.0	59.3	63.6
	*M*_R_, emu/g	18.7	23.5	28.3	30.8
	*M_R_/M_S_*	0.47	0.47	0.48	0.48
FO_32	*H*_C_, Oe	2520	2630	2780	3120
	*M*_S_, emu/g	37.6	44.2	49.9	52.4
	*M*_R_, emu/g	15.7	19.0	22.2	24.7
	*M_R_/M_S_*	0.42	0.43	0.44	0.47
FO_11	*H*_C_, Oe	2020	2330	2640	2990
	*M*_S_, emu/g	44.3	50.8	56.3	58.5
	*M*_R_, emu/g	18.2	21.2	24.7	27.9
	*M_R_/M_S_*	0.41	0.42	0.44	0.48

## Data Availability

The data presented in this study are available on request from the corresponding author.

## References

[1] McCallum R.W., Lewis L.H., Skomski R., Kramer M.J., Anderson I.E. (2014). Practical Aspects of Modern and Future Permanent Magnets. Annu. Rev. Mater. Res..

[2] Gorbachev E.A., Kozlyakova E.S., Trusov L.A., Sleptsova A.E., Zykin M.A., Kazin P.E. (2021). Design of Modern Magnetic Materials with Giant Coercivity. Russ. Chem. Rev..

[3] Odenbach S., Odenbach S. (2009). Colloidal Magnetic Fluids: Basics, Development and Application of Ferrofluids.

[4] Ohkoshi S., Namai A., Imoto K., Yoshikiyo M., Tarora W., Nakagawa K., Komine M., Miyamoto Y., Nasu T., Oka S. (2015). Nanometer-Size Hard Magnetic Ferrite Exhibiting High Optical-Transparency and Nonlinear Optical-Magnetoelectric Effect. Sci. Rep..

[5] Gorbachev E., Soshnikov M., Wu M., Alyabyeva L., Myakishev D., Kozlyakova E., Lebedev V., Anokhin E., Gorshunov B., Brylev O. (2021). Tuning the Particle Size, Natural Ferromagnetic Resonance Frequency and Magnetic Properties of ε-Fe_2_O_3_ Nanoparticles Prepared by a Rapid Sol–Gel Method. J. Mater. Chem. C.

[6] Gich M., Frontera C., Roig A., Taboada E., Molins E., Rechenberg H.R., Ardisson J.D., Macedo W.A.A., Ritter C., Hardy V. (2006). High- and Low-Temperature Crystal and Magnetic Structures of ε-Fe_2_O_3_ and Their Correlation to Its Magnetic Properties. Chem. Mater..

[7] Kubániová D., Brázda P., Závěta K., Kmječ T., Klementová M., Kohout J. (2019). Identification of Ferric Oxide Polymorphs in Nanoparticles Prepared by Sol-Gel Method and Maximization of ε-Fe_2_O_3_ Content. J. Magn. Magn. Mater..

[8] Pullar R.C. (2012). Hexagonal Ferrites: A Review of the Synthesis, Properties and Applications of Hexaferrite Ceramics. Prog. Mater. Sci..

[9] de Julian Fernandez C., Sangregorio C., de la Figuera J., Belec B., Makovec D., Quesada A. (2020). Topical Review: Progress and Prospects of Hard Hexaferrites for Permanent Magnet Applications. J. Phys. D Appl. Phys..

[10] Lisjak D., Mertelj A. (2018). Anisotropic Magnetic Nanoparticles: A Review of Their Properties, Syntheses and Potential Applications. Prog. Mater. Sci..

[11] Eliseev A.A., Eliseev A.A., Trusov L.A., Chumakov A.P., Boesecke P., Anokhin E.O., Vasiliev A.V., Sleptsova A.E., Gorbachev E.A., Korolev V.V. (2018). Rotational Dynamics of Colloidal Hexaferrite Nanoplates. Appl. Phys. Lett..

[12] Eliseev A.A., Trusov L.A., Anokhin E.O., Chumakov A.P., Korolev V.V., Sleptsova A.E., Boesecke P., Pryakhina V.I., Shur V.Y., Kazin P.E. (2022). Tunable Order in Colloids of Hard Magnetic Hexaferrite Nanoplatelets. Nano Res..

[13] Lukatskaya M.R., Trusov L.A., Eliseev A.A., Lukashin A.V., Jansen M., Kazin P.E., Napolskii K.S. (2011). Controlled Way to Prepare Quasi-1D Nanostructures with Complex Chemical Composition in Porous Anodic Alumina. Chem. Commun..

[14] Cao W., Yin S., Plank M., Chumakov A., Opel M., Chen W., Kreuzer L.P., Heger J.E., Gallei M., Brett C.J. (2021). Spray-Deposited Anisotropic Ferromagnetic Hybrid Polymer Films of PS-*b*-PMMA and Strontium Hexaferrite Magnetic Nanoplatelets. ACS Appl. Mater. Interfaces.

[15] Kushnir S.E., Kazin P.E., Trusov L.A., Tretyakov Y.D. (2012). Self-Organization of Micro- and Nanoparticles in Ferrofluids. Russ. Chem. Rev..

[16] Shirk B., Buessem W. (1971). Theoretical and Experimental Aspects of Coercivity versus Particle Size for Barium Ferrite. IEEE Trans. Magn..

[17] Kazin P.E., Trusov L.A., Kushnir S.E., Yaroshinskaya N.V., Petrov N.A., Jansen M. (2010). Hexaferrite Submicron and Nanoparticles with Variable Size and Shape via Glass-Ceramic Route. J. Phys. Conf. Ser..

[18] Trusov L.A., Babarkina O.V., Anokhin E.O., Sleptsova A.E., Gorbachev E.A., Eliseev A.A., Filippova T.V., Vasiliev A.V., Kazin P.E. (2019). Crystallization of Magnetic Particles in nNa_2_O-9SrO-6Fe_2_O_3_-8B_2_O_3_ (n = 1 and 4) Glasses. J. Magn. Magn. Mater..

[19] Kazin P.E., Trusov L.A., Zaitsev D.D., Tretyakov Y.D., Jansen M. (2008). Formation of Submicron-Sized SrFe_12−x_Al_x_O_19_ with Very High Coercivity. J. Magn. Magn. Mater..

[20] Trusov L.A., Sleptsova A.E., Duan J., Gorbachev E.A., Kozlyakova E.S., Anokhin E.O., Eliseev A.A., Karpov M.A., Vasiliev A.V., Brylev O.A. (2021). Glass-Ceramic Synthesis of Cr-Substituted Strontium Hexaferrite Nanoparticles with Enhanced Coercivity. Nanomaterials.

[21] Anokhin E.O., Trusov L.A., Kozlov D.A., Chumakov R.G., Sleptsova A.E., Uvarov O.V., Kozlov M.I., Petukhov D.I., Eliseev A.A., Kazin P.E. (2019). Silica Coated Hard-Magnetic Strontium Hexaferrite Nanoparticles. Adv. Powder Technol..

[22] Primc D., Makovec D. (2015). Composite Nanoplatelets Combining Soft-Magnetic Iron Oxide with Hard-Magnetic Barium Hexaferrite. Nanoscale.

[23] Primc D., Belec B., Makovec D. (2016). Synthesis of Composite Nanoparticles Using Co-Precipitation of a Magnetic Iron-Oxide Shell onto Core Nanoparticles. J. Nanopart. Res..

[24] Belec B., Dražić G., Gyergyek S., Podmiljšak B., Goršak T., Komelj M., Nogués J., Makovec D. (2017). Novel Ba-Hexaferrite Structural Variations Stabilized on the Nanoscale as Building Blocks for Epitaxial Bi-Magnetic Hard/Soft Sandwiched Maghemite/Hexaferrite/Maghemite Nanoplatelets with out-of-Plane Easy Axis and Enhanced Magnetization. Nanoscale.

[25] Haynes W.M., Lide D.R., Bruno T.J., Haynes W.M. (2016). CRC Handbook of Chemistry and Physics.

[26] Lutterotti L. (2010). Total Pattern Fitting for the Combined Size–Strain–Stress–Texture Determination in Thin Film Diffraction. Nucl. Instrum. Methods Phys. Res. Sect. B Beam Interact. Mater. At..

[27] Klinger M., Jäger A. (2015). Crystallographic Tool Box (CrysTBox): Automated Tools for Transmission Electron Microscopists and Crystallographers. J. Appl. Crystallogr..

[28] Klinger M. (2017). More Features, More Tools, More CrysTBox. J. Appl. Crystallogr..

[29] Obradors X., Solans X., Collomb A., Samaras D., Rodriguez J., Pernet M., Font-Altaba M. (1988). Crystal Structure of Strontium Hexaferrite SrFe_12_O_19_. J. Solid State Chem..

[30] Shannon R.D. (1976). Revised Effective Ionic Radii and Systematic Studies of Interatomic Distances in Halides and Chalcogenides. Acta Crystallogr. Sect. A.

[31] Makovec D., Belec B., Goršak T., Lisjak D., Komelj M., Dražić G., Gyergyek S. (2018). Discrete Evolution of the Crystal Structure during the Growth of Ba-Hexaferrite Nanoplatelets. Nanoscale.

[32] Makovec D., Dražić G., Gyergyek S., Lisjak D. (2020). A New Polymorph of Strontium Hexaferrite Stabilized at the Nanoscale. CrystEngComm.

[33] Markelova M., Nygaard R., Tsymbarenko D., Shurkina A., Abramov A., Amelichev V., Makarevich A., Vasiliev A., Kaul A. (2021). Multiferroic H-LuFeO_3_ Thin Films on (111) and (100) Surfaces of YSZ Substrates: An Experimental and Theoretical Study. ACS Appl. Electron. Mater..

[34] Momma K., Izumi F. (2011). VESTA3 for Three-Dimensional Visualization of Crystal, Volumetric and Morphology Data. J. Appl. Crystallogr..

[35] Gorbachev E.A., Trusov L.A., Kovalenko A.D., Morozov A.V., Kazin P.E. (2021). Sandwiched CoFe_2_O_4_/SrFe_11.5_Al_0.5_O_19_/CoFe_2_O_4_ Nanoparticles with Exchange-Coupling Effect. Nanoscale.

[36] Trusov L.A., Vasiliev A.V., Lukatskaya M.R., Zaytsev D.D., Jansen M., Kazin P.E. (2014). Stable Colloidal Solutions of Strontium Hexaferrite Hard Magnetic Nanoparticles. Chem. Commun..

[37] Zeng H., Li J., Liu J.P., Wang Z.L., Sun S. (2002). Exchange-Coupled Nanocomposite Magnets by Nanoparticle Self-Assembly. Nature.

[38] Kojima H. (1982). Chapter 5 Fundamental Properties of Hexagonal Ferrites with Magnetoplumbite Structure. Handbook of Ferromagnetic Materials.

[39] Cao L., Xie D., Guo M., Park H.S., Fujita T. (2007). Size and Shape Effects on Curie Temperature of Ferromagnetic Nanoparticles. Trans. Nonferrous Met. Soc. China.

[40] Guimarães A.P. (2009). Principles of Nanomagnetism.

[41] Nguyen T.N.A., Knut R., Fallahi V., Chung S., Le Q.T., Mohseni S.M., Karis O., Peredkov S., Dumas R.K., Miller C.W. (2014). Depth-Dependent Magnetization Profiles of Hybrid Exchange Springs. Phys. Rev. Appl..

